# The Case for Adopting a Multivariate Approach to Optimize Training Load Quantification in Team Sports

**DOI:** 10.3389/fphys.2017.01024

**Published:** 2017-12-12

**Authors:** Dan Weaving, Ben Jones, Kevin Till, Grant Abt, Clive Beggs

**Affiliations:** ^1^Institute for Sport, Physical Activity and Leisure, Leeds Beckett University, Leeds, United Kingdom; ^2^Leeds Rhinos Rugby League Club, Leeds, United Kingdom; ^3^School of Life Sciences, University of Hull, Kingston upon Hull, United Kingdom; ^4^Yorkshire Carnegie, Leeds, United Kingdom; ^5^The Rugby Football League, Leeds, United Kingdom

**Keywords:** multivariate analysis, global positioning systems (GPS), training load, reductionism, external training load, orthogonal analysis

Professional sports teams are investing substantial resources in monitoring the training load (TL) in their players in an attempt to achieve favorable training outcomes such as increases in performance and a reduction in negative outcomes such as injury. This investment is likely to increase as organizations explore the most recent developments in wearable technology that allow a wide variety of objective physiological and other measures to be collected concurrently and over long periods of time. The question of how all of this data can be used is one that many in our field are now asking (Foster et al., 2017). To answer this, we have to start with a definition of TL. Soligard et al. (2016) recently defined TL as:

“the sport and non-sport burden (single or multiple physiological, psychological, or mechanical stressors) as a stimulus that is applied to a human biological system (including subcellular elements, a single cell, tissues, one or multiple organ systems, or the individual)”

To quantify this construct, a common approach is to determine the ratio of a single measure across two moving-average time periods (e.g., acute- and chronic-training-load-ratio [A:C]). Suboptimal (either too high or low) TL is associated with an increased risk of injury (Hulin et al., 2016). However, while many TL methods (e.g., total distance, high-speed distance, session rating of perceived exertion [sRPE]) are collected, they are used individually as “predictor” variables in these analyses. Therefore, the initial consideration should be to determine the variable(s) that provide the most valid representation of the actual load imposed on each athlete.

Establishing the validity of a TL measure is typically examined through its agreement with a criterion which represents the true value. For example, the speed derived from a global positioning system (GPS) device is compared to that derived from a radar gun (Roe et al., 2016). In this instance, the confidence that the criterion measure represents the true value is high. In contrast, determining the validity of internal TL methods is problematic due to the limited physiological markers that are available in the field, and that there is no criterion method of measuring the internal TL. In addition, the definition highlighted previously (Soligard et al., 2016) demonstrates the complexity of the internal TL construct. Therefore, despite sRPE having been reported to correlate highly with Banister's training impulse (TRIMP) (*r* = 0.75) (Lovell et al., 2013) and Edward's TRIMP (*r* = 0.70) (Kelly et al., 2016) models, in these examples the shared variance is only 56 and 49%, respectively. This means that about half of the variance is unexplained. Are we therefore adopting a reductionist approach by assuming that by association, a single measure can capture the whole (true) internal TL imposed?

Physiological systems are complex, with many disparate factors affecting the outcomes of training. In essence, every bout of exercise/training imposes specific physiological, biomechanical, and psychological demands which vary not only with the prescribed “dose” (i.e., sets, repetitions, duration etc) but also with the mode (e.g., strength training vs. sport-specific training) of exercise (Soligard et al., 2016; Cardinale and Varley, 2017). Therefore, it is unlikely that a single independent variable will be able to capture this complexity and provide a valid measure of TL (either internal or external) and consequently, a holistic representation of TL has been suggested (Cardinale and Varley, 2017). By taking a univariate approach, we are in danger of omitting valuable information that could contribute to explaining the relationships between the imposed TL, and changes in fitness/performance/injury. For example, it is common practice to collect multiple TL variables concurrently. Recent investigations have shown that a single TL variable is unable to capture a meaningful proportion of the variance provided by multiple internal and external TL variables, which is exacerbated by the mode of training (e.g., technical-tactical, high-intensity-interval-training, sprint-training) (Weaving et al., 2014, 2017). Therefore, as the internal TL is governed largely by the external TL, external TL measures are likely to contribute “surrogate” information about the internal TL imposed and provide information that can also relate to training outcomes (Oxendale et al., 2016). In data science terms, the information contained collectively in, and between, these variables, has great potential to inform and optimize our understanding of training dose-response relationships. However, appropriately unlocking this information (without statistical/mathematical violation) can be difficult. As the variables associated with TL are often strongly correlated, multicollinearity (i.e., the degree to which variables are similar to one another) is frequently a problem. In addition, because player cohorts are small, it is often the case that the number of measured variables can exceed the number of players. As such, TL datasets can pose a considerable challenge when using traditional techniques such as logistic and multiple linear regression, thereby limiting their applicability when adopting multivariate (rather than univariate) TL analyses. However, through the use of dimension reduction techniques such as principal component analysis (PCA) (Weaving et al., 2014, 2017) and single value decomposition (SVD) (Till et al., 2016), which are immune to multicollinearity issues, it is possible to capture the complexity of a system in just a few orthogonal composite variables (i.e., variables that provide unique information). Because most of the variance in the system is captured in these orthogonal composite variables, it means that complex higher-dimensional systems can be represented on 2D and 3D scatter plots with minimal loss of information (Till et al., 2016). Furthermore, because the new variables are orthogonal it means that they are not correlated in any way, thus ensuring that they capture different attributes of the TL “system.” Single value decomposition and eigen-decomposition are at the heart of other useful data science techniques, such as partial least squares correlation analysis (PLSCA) (Beggs et al., 2016), which have great potential with respect to TL quantification. Rather than taking a conventional statistical approach, PLSCA utilizes the concept of shared information to gain new insights into the relationships between groups of variables (i.e., both predictor and response variables) in complex datasets. For example, using PLSCA, the relationship between multiple TL variables (e.g., total-distance, high-speed-distance, and s-RPE) and multiple “fatigue” variables can be investigated in a single analysis, allowing stronger inferences to made of the “dose-response” nature of these broad theoretical constructs that we wish to represent.

Despite the perceived increases in computational demands placed on practitioners, the authors feel that this multivariate approach warrants further investigation, at least initially in research, given the importance of TL measures in optimizing the preparation of team-sport players. It is then envisaged that this approach could be integrated into athlete monitoring software platforms to “combine” unique aspects of information provided by multiple TL variables. Although developing our understanding of what individual TL measures represent is important (i.e., validity), it is hoped that multivariate approaches will further develop our knowledge of the dose-response nature of TL monitoring with important training outcomes such as the changes in fitness, performance, and injury risk.

## Author contributions

DW, GA, and BJ: conceptualized the idea, wrote the introduction, and rationale to the commentary. DW and CB: wrote the discussion of the analysis approach to multivariate data. GA, KT, BJ, DW, and CB: drafted the manuscript, revised critically for important intellectual content.

### Conflict of interest statement

The authors declare that the research was conducted in the absence of any commercial or financial relationships that could be construed as a potential conflict of interest.
